# Malignant diagnosis and prognostic analysis of 89 GIST patients using preoperative FDG-PET

**DOI:** 10.1038/s41598-023-29038-5

**Published:** 2023-02-08

**Authors:** Kazuo Narushima, Kiyohiko Shuto, Shinichi Okazumi, Gaku Ohira, Mikito Mori, Koichi Hayano, Noriyuki Yanagawa, Hisahiro Matsubara

**Affiliations:** 1 Department of Surgery, Secomedic Hospital, Chiba, Japan; 2 Department of Frontier Surgery, Chiba University Graduate School of Medicine, Chiba, Japan; 3 Department of Surgery, Teikyo University Chiba Medical Center, 3426-3 Anesaki, Ichihara, Chiba 299-0111 Japan; 4 Department of Surgery, Toho University Sakura Medical Center, Chiba, Japan; 5 Department of Radiological Technology, Tsukuba International University, Ibaraki, Japan

**Keywords:** Surgical oncology, Diagnostic markers, Prognostic markers

## Abstract

There is no preoperative imaging accurately diagnose malignancy of gastrointestinal stromal tumor (GIST). To evaluate the usefulness of preoperative [^18^F]2-fluoro-2-deoxy-D-glucose positron emission tomography (FDG-PET) in the malignant diagnosis and prognostic analysis of GIST. Eighty-nine consecutive patients with GIST who underwent curative surgery were reviewed retrospectively. PET scan was performed within 2–3 weeks before surgery and maximum standardized uptake values (SUVmax) were assessed for GIST. The relationship between prognostic factors and prognosis of GIST and SUVmax were evaluated. Tumor size, mitotic count, and Ki-67 index showed significant positive correlations with the SUVmax. When the cutoff value was set as SUVmax 5.68, the accuracy was 86.5% for the high-risk group, 76.4% for the recurrence group, and 73.0% for the death group. The group with SUVmax ≥ 5.68 demonstrated a significantly lower 10-year relapse-free survival than the group with SUVmax < 5.68 (55.2% vs. 98.2%, *P* < 0.001), while the group with SUVmax ≥ 5.68 demonstrated a significantly lower 10-year overall survival than the group with SUVmax < 5.68 (68.0% vs. 97.6%, *P* < 0.001). In GISTs, FDG-PET is a very useful imaging marker for the diagnosis of malignant GISTs, such as those in high-risk and poor-prognosis groups.

## Introduction

Gastrointestinal stromal tumor (GIST) is the most common mesenchymal tumor of the gastrointestinal tract^1,2^. It is a relatively rare tumor, with an incidence of approximately 1 to 1.5 cases per 100,000 people per year^3^. The historical risk classification for GIST is the Fletcher classification, the National Institutes of Health (NIH) classification, using tumor size and mitotic count, which are important prognostic factors^4^. Recently, the usefulness of the modified Fletcher classification, which adds the two factors of tumor location and rupture, has been proposed^5^. Global clinical practice guidelines for GIST are provided by the National Comprehensive Cancer Network (NCCN)^6^ and the European Society for Medical Oncology (ESMO)^7^. Computed tomography (CT), endoscopic ultrasonography (EUS), magnetic resonance imaging (MRI), and [^18^F]2-fluoro-2-deoxy-D-glucose positron emission tomography (FDG-PET) are recommended for imaging of GIST. However, there is no imaging modalities that can provide a detailed diagnosis of mitotic count and risk classification before surgery.

The ability to diagnose high-risk and poor prognosis groups without surgery may contribute to the determination of indications for preoperative treatment and surgical procedures for reliable resection. There have been several reports of the diagnosis of risk classification using preoperative FDG-PET^8–12^. However, the number of cases was limited to approximately 30, and it is difficult to analyze each risk group in detail. In addition, there are still few reports examining the prognostic analysis of GIST in preoperative FDG-PET.

The purpose of this study was to evaluate the usefulness of preoperative FDG-PET in the malignant diagnosis and prognostic analysis of GIST using 89 cases of GIST.

## Results

### Patient characteristics

The clinical characteristics of 89 patients (41 women and 48 men) with GIST who underwent curative surgery are summarized in Table 1. The median age was 65 years (range, 38–83 years). The median tumor size was 3.5 cm (range, 1.5–30.0 cm). The median mitotic count was 4/HPF (range, 0–50/HPF). The median Ki-67 was 5.0% (range, 1.0–40.0%). Tumor locations were as follows: 2 patients, esophagus; 68 patients, stomach; 9 patients, small bowel; and 10 patients, colorectum. Type of surgery was as follows: 68 patients, partial resection; 17 patients, gastrectomy; 4 patients, ileocecal resection, low anterior resection, and abdominoperineal resection. Modified Fletcher risk classifications were as follows: 9 patients, very low risk; 40 patients, low risk; 17 patients, intermediate risk; and 23 patients, high risk. The median postoperative follow-up duration was 2336 days (range, 391–6285 days). Ten patients developed recurrence and 7 patients died.

**Table 1:** Patient characteristics.

Variables	
Sex	
Male	48 (53.9%)
Female	41 (46.1%)
Age, years	65 (38–83)
Size, cm	3.5 (1.3–30.0)
Mitotic count, /HPF	4 (0–50)
Ki-67, %	5.0 (1.0–40.0)
Tumor location	
Esophagus	2 (2.3%)
Stomach	68 (76.4%)
Small bowel	9 (10.1%)
Colorectum	10 (11.2%)
Type of surgery	
Partial resection	68 (76.4%)
Gastrectomy	17 (19.1%)
ICR, LAR, APR	4 (4.5%)
Risk classification (modified Fletcher)	
Very low	9 (10.1%)
Low	40 (44.9%)
Intermediate	17 (19.1%)
High	23 (25.9%)
Postoperative observation period, days	2336 (391–6285)
Recurrence	10
Death	7

HPF, high power field; ICR, ileocecal resection; LAR, low anterior resection; APR, abdominoperineal resection.

### Representative cases

**Figure 1:**
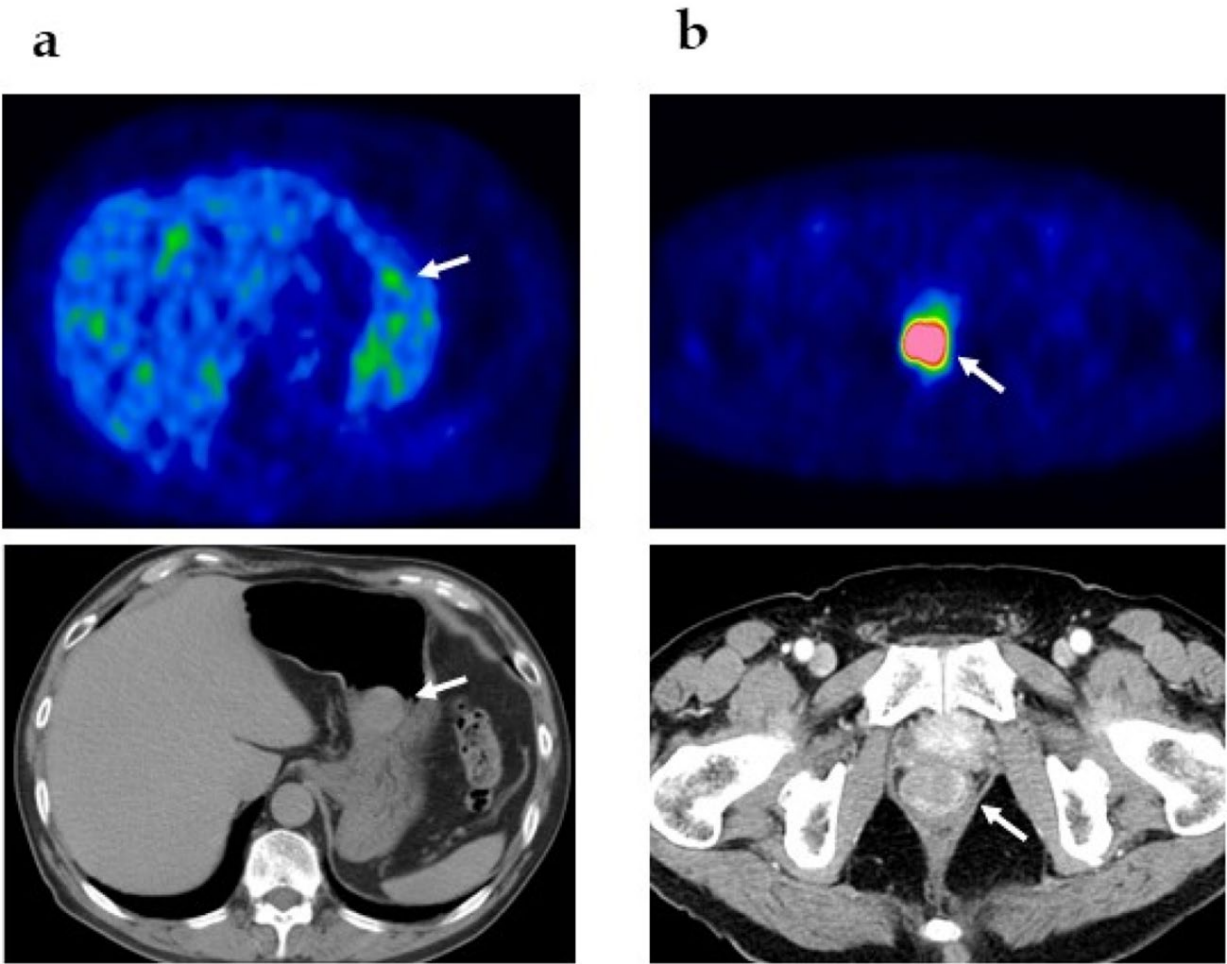
Representative cases. (**a**) Low-risk case in the stomach with a tumor size of 3.3 cm, mitosis 3/HPF, and SUVmax 4.58. (**b**) High-risk case in the rectum with a tumor size of 3.0 cm, mitosis 26/HPF, and SUVmax 20.68. Arrows indicate tumors.

Representative cases of FDG-PET images for the low- and high-risk groups are shown in Fig. 1. A low-risk case in the stomach showed a tumor size of 3.3 cm, mitosis 3 HPF, and SUVmax 4.58 (Fig. 1a), while a high-risk case in the rectum showed a tumor size of 3.0 cm, mitosis 26 HPF, and SUVmax 20.68 (Fig. 1a).

### Correlation between SUVmax and tumor size of GIST

**Figure 2:**
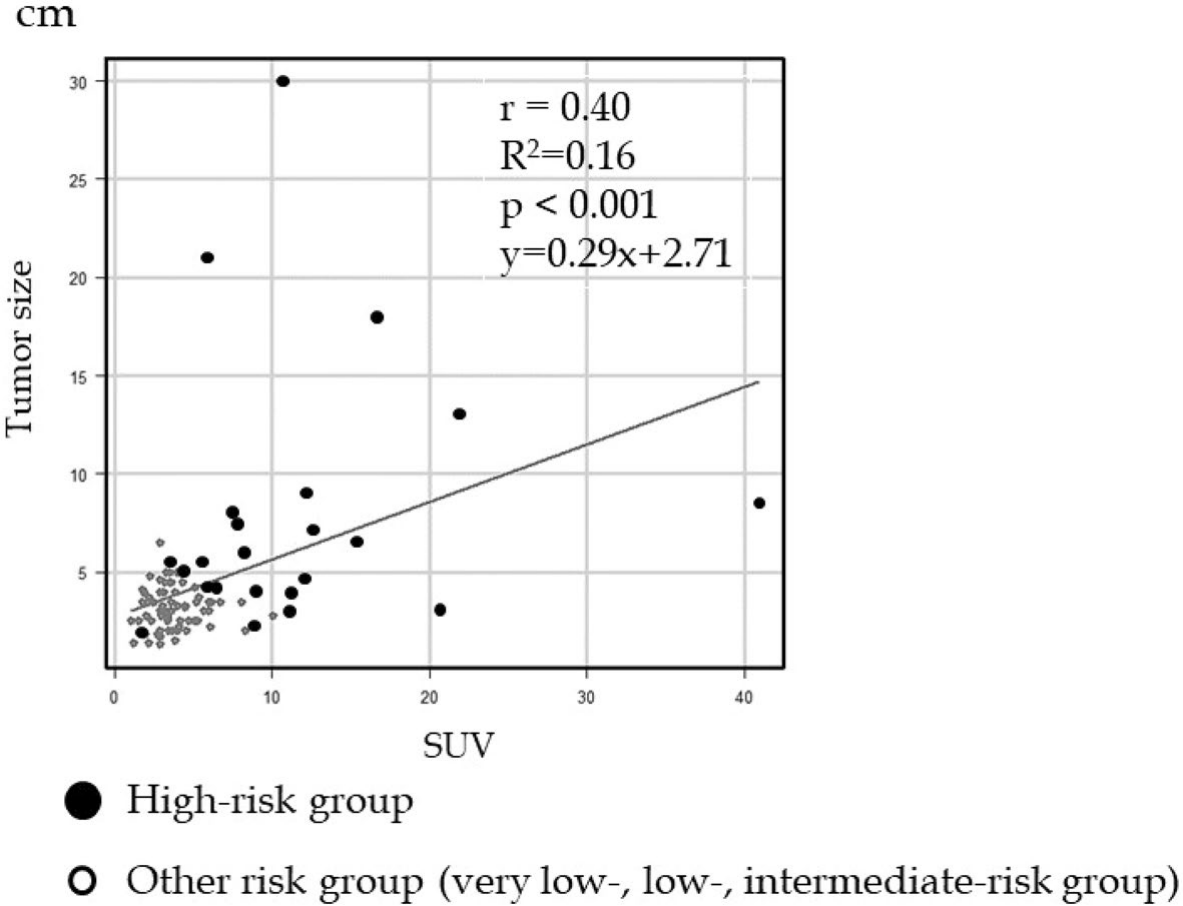
Correlation between SUVmax and tumor size of GIST. There was a positive linear relationship between SUVmax and tumor size. In GISTs with a tumor size of 5 cm or less, 10 patients were in the high-risk group and 8 had SUV ≥ 5.68.

The correlation between the SUVmax and tumor size is shown in Fig. 2. A positive linear relationship between the SUVmax and tumor size (Y = 2.71 + 0.29 X, R^2^ = 0.16, correlation coefficient = 0.40, *P* < 0.001) were observed. In GISTs with a tumor size of 5 cm or less, 10 patients were in the high-risk group, and 8 had SUVmax ≥ 5.68.

### Correlation between the SUVmax and mitotic count and Ki-67 of GIST

**Figure 3:**
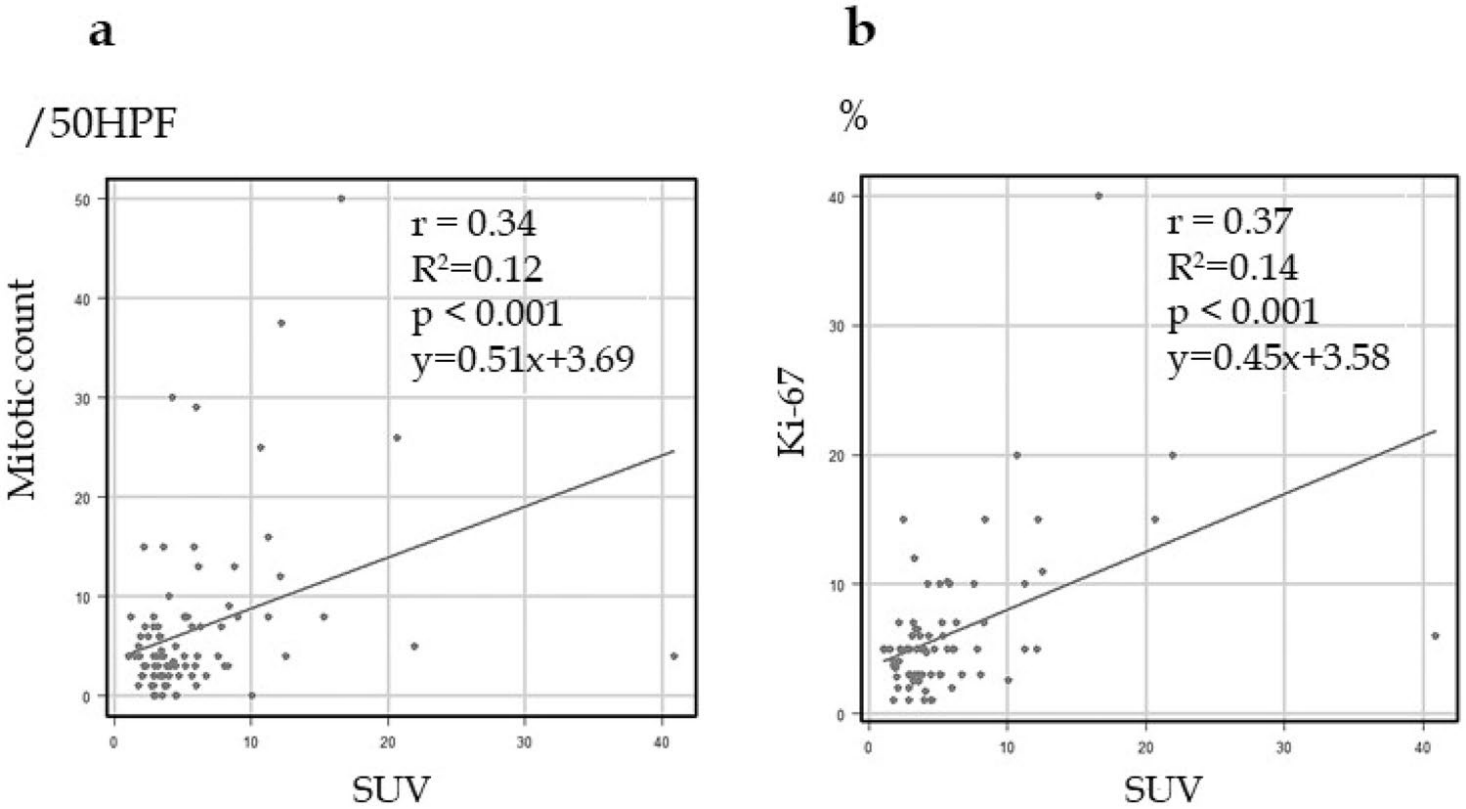
Correlation between SUVmax and mitotic count and Ki-67 of GIST. There was a positive linear relationship between SUVmax and mitotic count and Ki-67. (**a**) Mitotic count. (**b**) Ki-67.

The correlation between the SUVmax and mitotic count and Ki-67 is shown in Fig. 3. A positive linear relationship between the SUVmax and mitotic count (Y = 3.69 + 0.51*X*, R^2^ = 0.12, correlation coefficient = 0.34, *P* < 0.001; Fig. 3a) and a positive relationship between the SUVmax and Ki-67 (Y = 3.58 + 0.45*X*, R^2^ = 0.14, correlation coefficient = 0.37, *P* < 0.001; Fig. 3b) was observed.

### Relationship between the SUVmax and risk groups of GIST

**Figure 4:**
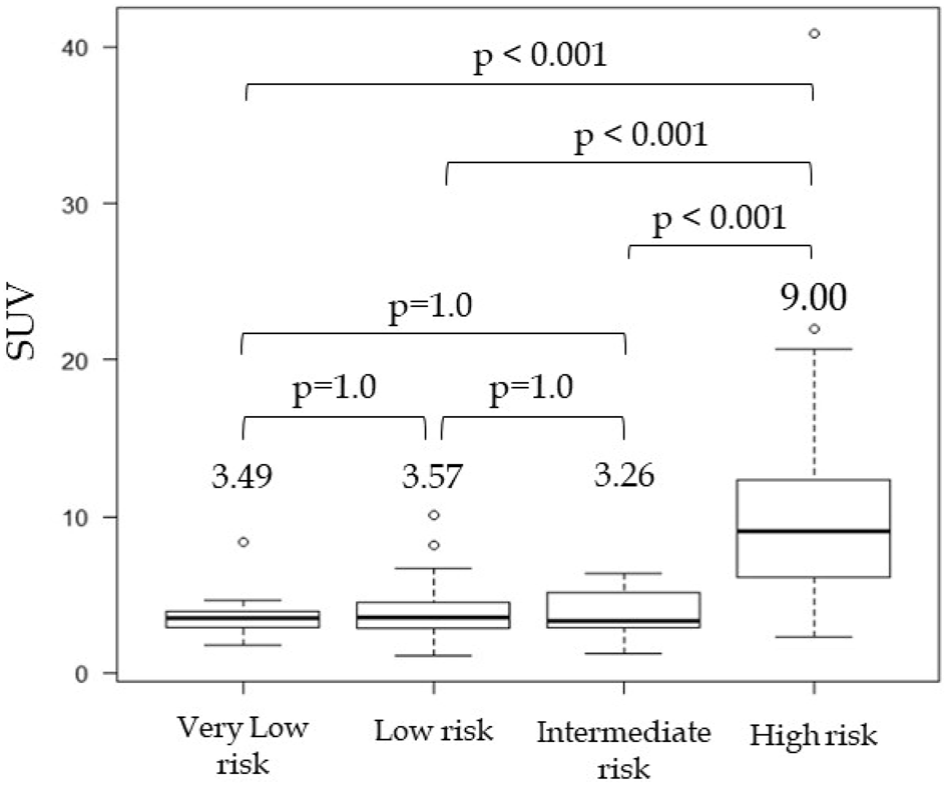
Relationship between SUVmax and risk groups of GIST. Only the high-risk group with the highest malignancy of GIST had a significantly higher SUVmax. SUVmax showed no statistically significant difference between the other risk groups or the very low-, low-, and intermediate-risk groups.

The relationship between the SUVmax and risk groups of GIST is shown in Fig. 4. The median SUVmax in risk groups was 3.49, 3.57, 3.26, and 9.00 for the very low-risk, low-risk, intermediate-risk, and high-risk groups, respectively (*P* = 1.0 for the difference between the very low-risk and low-risk groups; *P* = 1.0 for the low-risk and intermediate-risk groups; *P* = 1.0 for the very low-risk and intermediate-risk groups; *P* < 0.001 for the very-low risk and high-risk groups; *P* < 0.001 for the low-risk and high-risk groups; and *P* < 0.001 for the intermediate-risk and high-risk groups).

### Diagnostic ability for high-risk group of GIST

**Figure 5:**
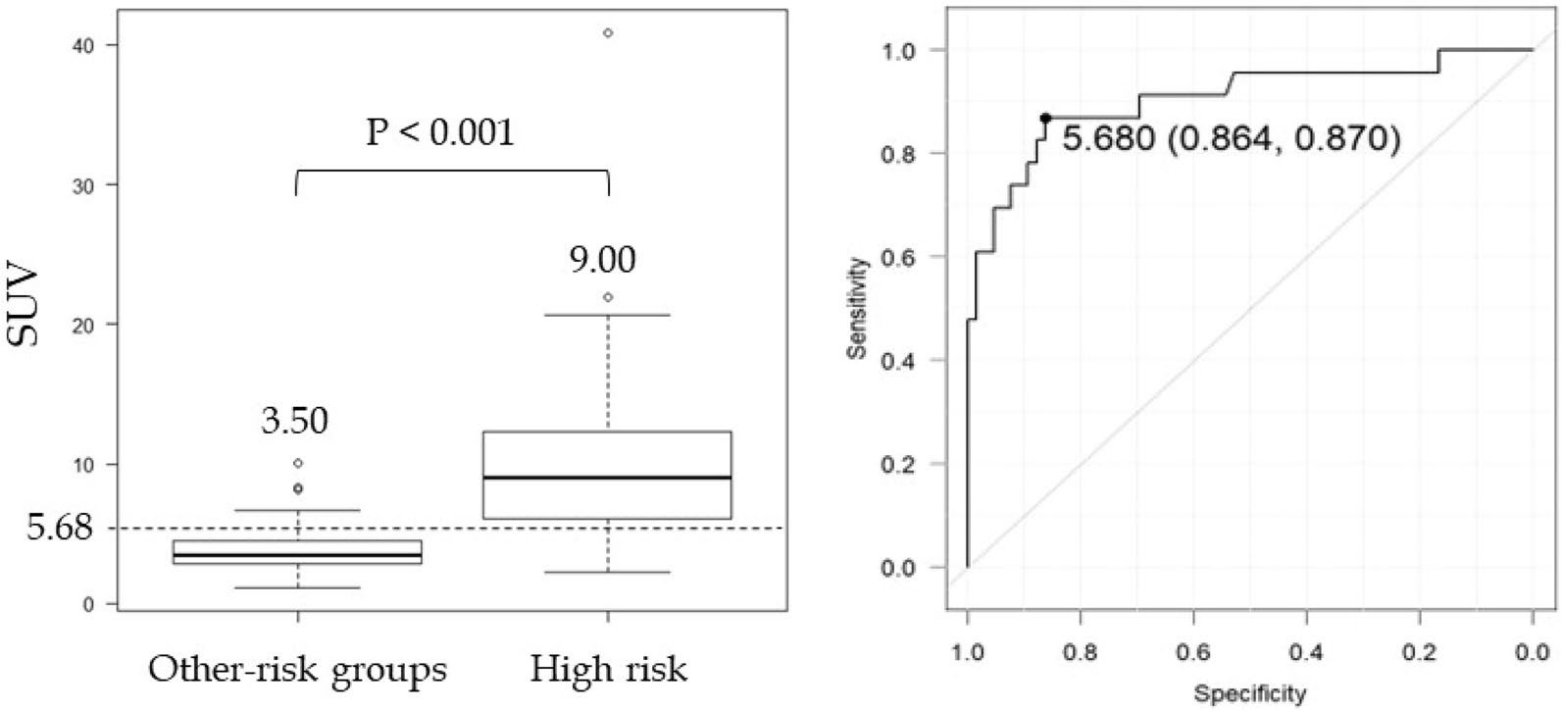
Relationship between the other risk groups and the high-risk group of GIST. There was also a significant difference in SUVmax between the other risk groups and the high-risk group (*P* < 0.001). The cutoff value for high-risk group diagnosis using ROC curves was SUVmax ≥ 5.68.

The relationship between the high-risk group and other groups of GIST is shown in Fig. 5 and Table 2. In a comparison of the high-risk and other risk groups, median SUVmax was 9.00 and 3.50 for the high-risk group and other risk groups, respectively (*P* < 0.001 for the difference between the high risk and other risk groups). When the cutoff value was set as SUVmax 5.68 for the high-risk group, the area under the curve (AUC) was 0.905 and the sensitivity, specificity, and accuracy were 87.0%, 86.4%, and 86.5%, respectively. When the cutoff value was set as SUVmax 5.68 for the high-risk group in GISTs with a tumor size of 5 cm or less, the sensitivity, specificity, and accuracy were 80.0%, 86.2%, and 85.3%, respectively. When the cutoff value was set as SUVmax 5.68 for the high-risk group in GISTs with a tumor size of 5 cm more than, the sensitivity, specificity, and accuracy were 92.3%, 100.0%, and 92.9%, respectively.

**Table 2:** Diagnostic accuracy of high-risk, recurrence, and death groups using a cutoff of SUVmax ≥ 5.68.

High-risk group	Sensitivity (%)	Specificity (%)	PPV (%)	NPV (%)	Accuracy (%)
All cases	87.0 (20/23)	86.4 (57/66)	69.0 (20/29)	95.0 (57/60)	86.5 (77/89)
Tumor size ≤ 5	80.0 (8/10)	86.2 (56/65)	47.1 (8/17)	96.6 (56/58)	85.3 (64/75)
Tumor size 5 <	92.3 (12/13)	100.0 (1/1)	100.0 (12/12)	50.0 (1/2)	92.9 (13/14)
Prognosis (all cases)					
Recurrence group					
High-risk group	90.0 (9/10)	82.3 (65/79)	39.1 (9/23)	98.5 (65/66)	83.1 (74/89)
SUVmax ≥ 5.68	90.0 (9/10)	74.7 (59/79)	31.0 (9/29)	98.3 (59/60)	76.4 (68/89)
Death group					
High-risk group	71.4 (5/7)	78.0 (64/82)	21.7 (5/23)	97.0 (64/66)	77.5 (69/89)
SUVmax ≥ 5.68	85.7 (6/7)	72.0 (59/82)	20.7 (6/29)	98.3 (59/60)	73.0 (65/89)

PPV, positive predictive value; NPV, negative predictive value.

### Relationship between the SUVmax and prognosis of GIST

**Figure 6:**
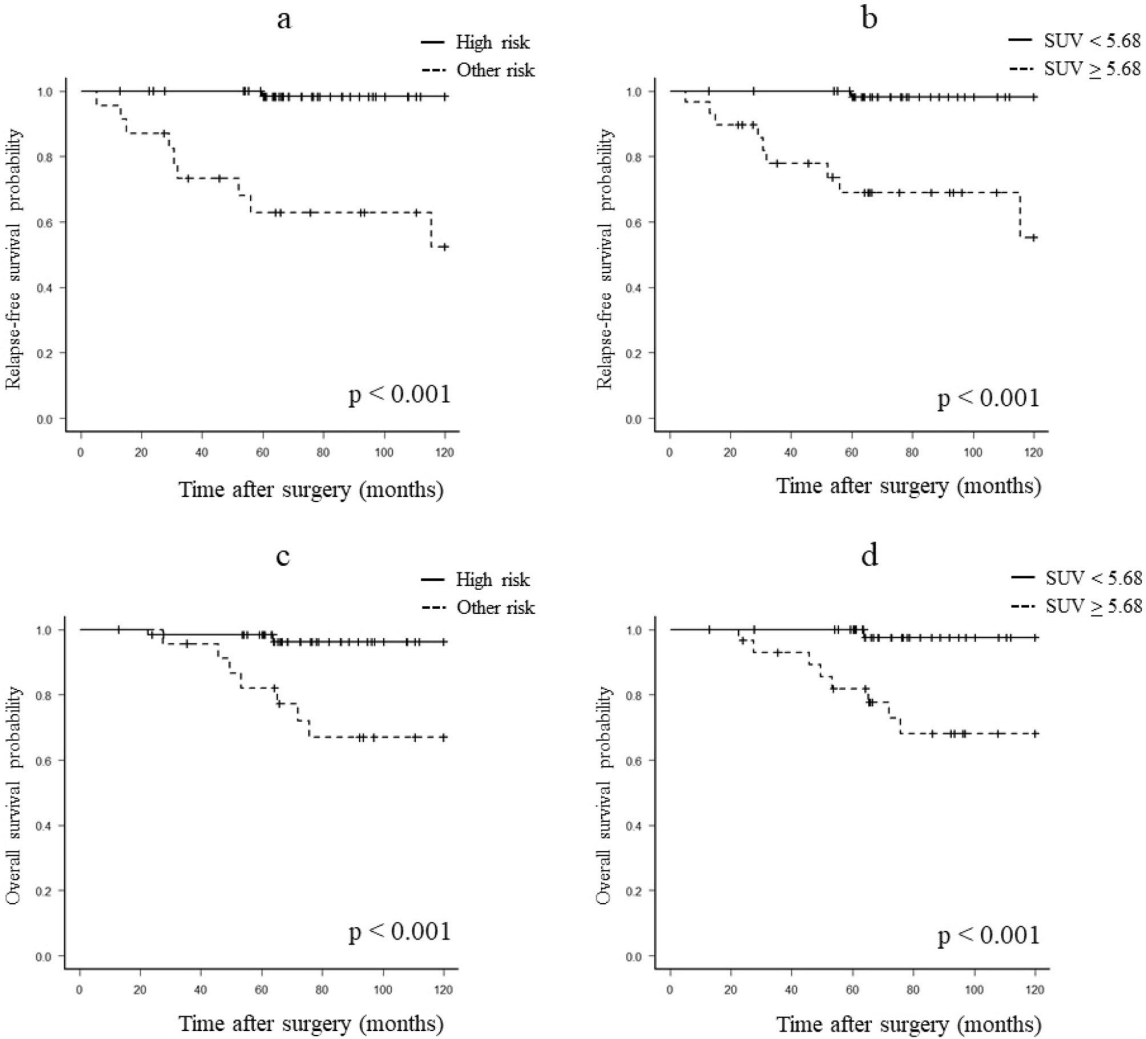
Survival curves of relapse-free survival and overall survival. (**a**) Relapse-free survival of patients with high risk- and other-risk groups using the log-rank test (*P* < 0.001). (**b**) Relapse-free survival of patients with SUVmax ≥ 5.68 and < 5.68 using the log-rank test (*P* < 0.001). (**c**) Overall survival of patients with high risk- and other-risk groups using the log-rank test (*P* < 0.001). (**d**) Overall survival of patients with SUVmax ≥ 5.68 and < 5.68 using the log-rank test (*P* < 0.001).

The diagnostic ability for the recurrence and death groups is shown in Table 2. The diagnostic accuracy of recurrence group in the high-risk group was the sensitivity, specificity, and accuracy were 90.0%, 82.3%, and 83.1%, respectively. When the cutoff value was set as SUVmax 5.68 for the recurrence group, the sensitivity, specificity, and accuracy were 100.0%, 75.0%, and 77.5%, respectively. The diagnostic accuracy of death group in the high-risk group was the sensitivity, specificity, and accuracy were 71.4%, 78.0%, and 77.5%, respectively. When the cutoff value was set as SUVmax 5.68 for the death group, the sensitivity, specificity, and accuracy were 85.7%, 72.0%, and 73.0%, respectively. The survival curves of the recurrence and death groups are shown in Fig. 6. High-risk group demonstrated significantly lower 10-year RFS than other risk groups (52.3% vs. 98.3%, *P* < 0.001) (Fig. 6a). Likewise, the group with SUVmax ≥ 5.68 demonstrated significantly lower 10-year RFS than the group with SUVmax < 5.68 (55.2% vs. 98.2%, P < 0.001) (Fig. 6b). High-risk group demonstrated significantly lower 10-year OS than other risk groups (66.9% vs. 96.2%, *P* < 0.001) (Fig. 6c). Likewise, the group with SUVmax ≥ 5.68 demonstrated significantly lower 10-year OS than the group with SUVmax < 5.68 (68.0% vs 97.6%, *P* < 0.001) (Fig. 6d).

## Discussion

Many studies on the usefulness of FDG-PET for GIST have been reported. Among them, five have attempted a detailed diagnosis of GIST malignancy using preoperative FDG-PET^8–12^. These reports showed that the SUVmax is positively correlated with tumor size, mitotic count, and Ki-67, and that high-risk and intermediate-risk patients can be diagnosed above a certain cutoff value. However, these five papers analyzed only 10 to 30 cases, with a maximum of 32 cases and a few cases each in the high- and intermediate-risk groups. It seemed difficult enough to compare the four risk groups or to consider them against a relatively high-risk group. In this study, a total of 89 cases, including previously unreported esophageal GISTs, were examined. The number of patients in each risk group was relatively sufficient: 23 in the high-risk group, 17 in the intermediate-risk group, 40 in the low-risk group, and 9 in the very low-risk group.

First, correlations between SUVmax and various important quantitative GIST factors of tumor size, mitotic count, and Ki-67 were examined. Tumor size and mitotic count are components that determine the risk classification of GIST. Ki-67 is a cell proliferation marker that has been reported to correlate with mitotic count and prognosis in GIST^13–15^. It has been reported that tumor size, mitotic count, and Ki-67 are positively correlated with SUVmax, and thus the reason for the higher SUVmax in the high-risk group^8–12^. Similarly, in the present study, all factors showed a positive correlation with SUVmax, but the correlation was not as strong. It seemed difficult to accurately diagnose these factors using FDG-PET. The reason why only SUVmax was significantly higher in the high-risk group was probably due to the combined factors of tumor size, mitotic count, and Ki-67.

Next, the relationship between modified Fletcher classification risk groups and FDG uptake was examined. Only the high-risk group with the highest malignancy of GIST had a significantly higher SUVmax. It has been reported that SUVmax is higher in the high-risk group of GIST^8,11^. The SUVmax showed no statistically significant differences between the other risk groups and the very low-, low-, and intermediate-risk groups, and it seemed difficult to distinguish using FDG-PET. This is the first report to show no statistically significant difference between the SUVmax of the other risk groups. When the cutoff value was set at an SUVmax ≥ 5.68, the diagnostic ability of the high-risk group was highly accurately at approximately 86%. The accuracy was also high in GIST with a tumor diameter of 5 cm or less, which was difficult to diagnose in the high-risk group, with a diagnostic accuracy of approximately 85%. It appears that a high SUVmax in preoperative GIST is predictive of high-grade malignancy, and SUVmax is a useful and important imaging marker for diagnosing high-risk groups. Recent studies have shown risk classification diagnosis by preoperative PET markers other than SUVmax. It has been shown that 18F-FDG uptake correlates with tumor size, tumor risk grade, and expression levels of glucose transporter 1 (GLUT1), hexokinase 1 (HK1), and lactate dehydrogenase A (LDHA), with GLUT1, HK1, pyruvate kinase (PKM2), and LDHA expression increasing with higher tumor risk grade^16^. It may be difficult to perform all of these immunostains in general practice. It has also been shown that ring-shaped uptake of GISTs correlates with high-risk GISTs^17^ while not a semi-quantitative test.

In addition, the relationship between the prognosis of GIST and SUVmax was examined. Using a cutoff value of SUVmax ≥ 5.68, which diagnoses the recurrence group, the diagnostic ability of the recurrence group was highly accurate, at nearly 80%, and GIST patients with an SUVmax ≥ 5.68 had significantly poorer RFS than GIST patients with SUVmax < 5.68. Using a cutoff value of SUVmax ≥ 5.68, which diagnosed the death group, the diagnostic ability of the death group was highly accurate, at over 70%, and GIST patients with SUVmax ≥ 5.68 had significantly poorer OS than GIST patients with SUVmax < 5.68. The prognosis of GIST risk groups is very poor for the high-risk group regarding RFS^18^. SUVmax ≥ 5.68 may be one basis for being able to diagnose the high-risk group. Recent studies have shown prognostic markers for GIST on preoperative FDG-PET other than SUVmax. Independent prognostic factors for RFS of GIST have been shown to be ring-shaped uptake^17^ and metabolic tumor volume (MTV) and total lesion glycolysis (TLG)^19,20^. However, SUVmax is an excellent semi-quantitative PET indicator that is general, quick, and roboust. Therefore, this method might be enough to predict higher risk patients.

Clinical applications of the results of this study include the following. First is the application of FDG-PET in the diagnosis of the high-risk group for preoperative GISTs. Risk classification of GISTs requires measurement of the mitotic count. However, it is difficult to accurately diagnose the mitotic count of GISTs preoperatively because its measurement requires total tumor removal and retrieval. Therefore, it is usually difficult to diagnose the risk classification of GISTs before treatment. Even in international GIST practice guidelines such as the NCCN and ESMO, no imaging test has been proposed that can diagnose the mitotic count and risk classification of GISTs. Based on the results of this study, we propose that FDG-PET is a useful imaging test that can diagnose high-risk groups of GISTs with high accuracy. GISTs with SUVmax ≥ 5.68 on preoperative FDG-PET would require surgery with adequate resection margins. It is also useful in GISTs with tumor diameters of 5 cm or less, which are difficult to diagnose in high-risk groups. Second is the application of FDG-PET in preoperative adjuvant chemotherapy of GISTs, whereby FDG-PET may be able to aid in the selection of the indication for preoperative adjuvant chemotherapy. Various prospective and retrospective studies of preoperative adjuvant chemotherapy for GISTs have been conducted^21,22^, but the goal has primarily been to reduce the size of large tumors for surgery. If preoperative adjuvant chemotherapy to improve the prognosis of GIST is established as a standardized treatment in the future, FDG-PET may become an imaging marker to select high-risk and poor-prognosis groups for preoperative adjuvant chemotherapy. The ability to diagnose prognostic groups of GISTs by FDG-PET could have a variety of therapeutic benefits that would be of great benefit to GIST patients. For these reasons, we believe that this study has very important clinical implications.

This study had several study limitations. First, Because GISTs are relatively rare tumors, there was a small sample size of patients for study. In addition, the prognosis for GIST outside of the high-risk group was relatively good, so the number of cases for prognostic analysis was small. Second, because this study was retrospective, there may be selection bias regarding the study subjects. Third, the Advance NXi PET scanner is not integrated with CT, making it difficult to measure these more advanced PET metrics. The localization of low FDG avid GISTs was identified by comparison with separately performed CT imaging.

## Conclusion

Preoperative FDG-PET is a very useful potential imaging marker for the identification and stratification of high-risk and poor-prognosis groups in patients with malignant GISTs.

## Materials and methods

### Patients

From June 2003 to July 2014, 89 consecutive patients with GIST who underwent curative surgery at the Department of Frontier Surgery, Graduate School of Medicine, Chiba University, were reviewed retrospectively. Preoperative investigation was performed using endoscopy, CT, endoscopic ultrasound (EUS), radiography of the upper digestive tract, and FDG-PET. In the analysis, there were no patients who was less than 20 years of age, underwent emergency surgery, and had other malignancies. All patients underwent complete tumor resection, and risk classification of GIST was performed using the Modified Fletcher classification^7^ after pathological diagnosis. All Patients were followed up using CT and endoscopy. Patients in the high-risk group received adjuvant chemotherapy.

### FDG PET imaging

Within 3 weeks before surgery, PET was performed using a single whole-body PET system (Advance NXi; GE Medical Systems, Milwaukee, WI, USA). After an injection of 370 MBq (10 mCi) FDG tracer, scanning was initiated from the top of the brain to the upper thigh with a rotating external source by the simultaneous 4-min emission and 2-min transmission method. Attenuation-corrected transaxial images were reconstructed by the ordered subsets expectation maximization algorithm into 128 × 128 matrices, a 55-cm field of view, and a 4.25-mm section thickness. The region of interest (ROI) was measured as the area of maximum FDG uptake encompassing the entire lesion on the transaxial planes. If the FDG uptake of the lesion was not clear, the ROI was measured as the location of the tumor detected on the CT image. Maximum standardized uptake values (SUVmax), which are convenient semiquantitative methods and most widely used, were assessed for FDG uptake by the lesion.

### Surgery and pathological diagnosis

All patients underwent radical tumor resection. Tumor specimens obtained by surgical resection were fixed in 10% formaldehyde, embedded in paraffin, and stained with hematoxylin and eosin for pathological evaluation of the mitotic index, which indicates the number of mitotic cells per 50 high-power fields (HPFs). Immunohistochemical staining was performed using the avidin–biotin complex method with a kit and the following antibodies: c-kit, CD34, smooth muscle actin, S-100, and Ki-67.

### Postoperative follow-up and treatment

Patients in the high-risk group received imatinib (400 mg per day) as adjuvant chemotherapy. Patients were followed up every 3–6 months for the first 5 years and then on an annual basis. Contrast-enhanced CT scans of the chest and abdomen were performed every 6 months and endoscopy was performed yearly. Patients who relapsed received chemotherapy. Chemotherapy consisted of imatinib (400 mg per day) for first-line therapy, sunitinib (50 mg per day for 4 weeks followed by a 2-week rest) for second-line therapy, and regorafenib (80 mg per day for 3 weeks followed by a 1-week rest) for third-line therapy.

### Statistical analyses

All values were calculated as the median. Statistical analyses were performed using the EZR software program (Saitama Medical Center, Jichi Medical University, Saitama, Japan)^23^, with *P*-values of less than 0.05 considered statistically significant. The relationship between prognostic factors of GIST (tumor size, mitotic index, and Ki-67) and SUVmax were evaluated using the non-parametric Spearman's rank correlation test and linear regression. Comparisons between the SUVmax of risk groups were performed by multiple comparisons of Kruskal–Wallis test and Bonferroni correction. Comparisons between the SUVmax of the high-risk group and other groups were compared by performing the Mann–Whitney U test. Cutoff values to diagnose the high-risk group were determined using the receiver operating characteristic (ROC) curve. The best cutoff points for balancing the sensitivity and specificity of a test are the point on the curve closest to the (0, 1) point. Optimal sensitivity and specificity are defined as those yielding the minimal value for (1 − sensitivity)^2^ + (1 − specificity)^2^^24^. The overall survival (OS) time was defined as the period from the date of surgery to the date of death or the last follow-up. The relapse-free survival (RFS) time was defined as the period from the day of surgery to the day of recurrence. OS and RFS curves were generated using the Kaplan–Meier method.

### Ethical approval

All enrolled patients received explanations, upon which they provided written informed consent regarding this clinical study. Our study was conducted in accordance with the principles outlined in the 1964 Declaration of Helsinki and its later amendments. All procedures and subsequent analyses were performed with the approval of the Institutional Review Boards of Chiba University (reference number: 1079).

## Data Availability

Technical appendix, statistical code, and dataset available from the corresponding author at kshuto@med.teikyo-u.ac.jp. No additional data are available.
